# A study on the association between accessory maxillary ostium and maxillary sinus mucosal thickening using cone beam computed tomography

**DOI:** 10.1186/s13005-021-00284-0

**Published:** 2021-07-14

**Authors:** Shishir Shetty, Saad Wahby Al Bayatti, Natheer Hashim Al-Rawi, Rani Samsudin, Hesham Marei, Raghavendra Shetty, Hossam Abdelatty Abdelmagyd, Sesha Reddy

**Affiliations:** 1 Department of Oral and Craniofacial Health Sciences, College of Dental Medicine, University of Sharjah, Sharjah, United Arab Emirates; 2 College of Dentistry, Gulf Medical University, Ajman, United Arab Emirates; 3 College of Dentistry, Ajman University, Ajman, United Arab Emirates

**Keywords:** Maxillary sinus, Nasal cavity, CBCT, Sinusitis, Mucosal thickening, Accessory maxillary ostia

## Abstract

**Background:**

Accessory maxillary ostium (AMO) has a major role to play in the aetiology of maxillary sinusitis. Mucosal thickening is one of the key radiographic features of chronic maxillary sinusitis. The aim of this study was to identify the location of the AMOs and investigate the association between Mucosal Thickening [MT] and AMO using Cone Beam Computed Tomography [CBCT].

**Methods:**

CBCT scans of 400 maxillary sinuses from the records of 200 patients who seeked various dental treatments at the Thumbay Dental Hospital, Gulf Medical University, Ajman, United Arab Emirates were evaluated. The incidence, anatomical position and maximal length of accessory maxillary ostia (AMO) in the maxillary antrum were reviewed using CBCT by two examiners. The association between MTs and AMOs were also analysed.

**Results:**

Among the 200 CBCT scans, 131 belonged to male patients and 69 scans belonged to female subjects within the age group of 18–65 years (mean age 41.32 years). AMOs were found in 142 maxillary antra (35.5 %). The inter-observer reliability for using CBCT to detect AMO was (k = 0.83). There was no significant difference in the frequency of AMOs when the age (*P* = 0.19) and gender (*P* = 0.54) distribution were considered. Sinuses with AMOs, showed significantly greater frequency of MTs (*p* = 0.001). AMOs with maximal length of less than 1mm were most commonly observed (51.40 %). AMOs with larger greater maximal length were associated with higher degrees of MT. The location of the AMOs, were not affected by the degree of MT.

**Conclusions:**

The study demonstrates a clear association between degree of MT and occurrence of AMO in the maxillary sinus. However, the location of the AMO is independent of the degree of the MT. There is a greater probability of finding an AMO in the maxillary sinus if the MT in the sinus is more than 3 mm.

**Supplementary Information:**

The online version contains supplementary material available at 10.1186/s13005-021-00284-0.

## Background

Chronic maxillary sinusitis [CMS] is one of the most common disease conditions that takes a patient to the otolaryngologist [1]. CMS has a multifactorial etiology ranging from bacterial infection and allergy to nasal anatomical variations [1]. Accessory maxillary ostium [AMO] is believed to play a role in CMS aetiology [2]. Some researchers believe that mucous that has been drained through primary ostium may re-enter into the maxillary sinus through the AMO leading to “mucus recirculation” [3, 4]. However other researchers state that AMO develops following an acute maxillary sinusitis. Therefore, whether AMO is the cause or the result of maxillary sinusitis, is still uncertain. It is also debatable whether AMO is congenital or acquired [5]. There has been a significant correlation between AMO and CMS in previous studies carried out using computed tomography [6, 7].

Symptoms of maxillary sinusitis may sometimes appear in the maxillary dento-alveolar region and Cone Beam Computed tomography [CBCT] may be performed as part of dental investigation. This increases the possibility for a dental surgeon, finding an AMO during evaluation of the CBCT scans. While Computed Tomography [CT] offers superior image, quality compared to CBCT, the latter exposes the patient to a substantially lower radiation dose [8]. The aim of this study was to identify the location of the AMOs and investigate the association between Mucosal Thickening [MT] and AMO.

## Material and methods

CBCT images from 200 patients (*n* = 400 maxillary sinuses) who underwent maxillofacial scans at Thumbay Dental Hospital, Gulf Medical University, Ajman, United Arab Emirates [UAE] were evaluated in this retrospective study. Human Ethical Approval was obtained from the Institutional Review Board of the Gulf Medical University (Ref. no. INT/COD/FR/006-2020). The CBCT image examinations of the sino-nasal variants were performed using a ProMax 3D Mid machine (Planmeca, Helsinki, Finland), operated at 90 kVp and 10 mA with a 9 × 16 cm field of view. Assessment of CBCT scans was performed directly on a 1920 × 1080 pixel and 23-inch DELL monitor screen. The voxel edge length was 0.2 mm.

CBCT scans of male and female subjects within the age range of 18 to 65 years were included in the study. The maxillary dental status was classified as dentate, partially edentulous, and completely edentulous. The classification was based on the presence or absence of teeth distal to maxillary canine till the maxillary third molar bilaterally.

CBCT scans of improper quality were excluded from the study, these included streak artifacts (*n* = 4), incomplete images (*n* = 2). Exclusion criteria for patients in this study were for subjects who had a history of midfacial trauma (*n* = 2), tumour (*n* = 1), cleft palate (*n* = 1), and syndromes effecting the midface (*n* = 1). All selected CBCT scans of the study were evaluated by two radiologists (A) and (B) with more than ten years of clinical experience in dento-maxillofacial radiology. In case of inter-observer disagreement, a third oral radiologist (C) with equivalent clinical experience was consulted for the final decision.

**Fig. 1:**
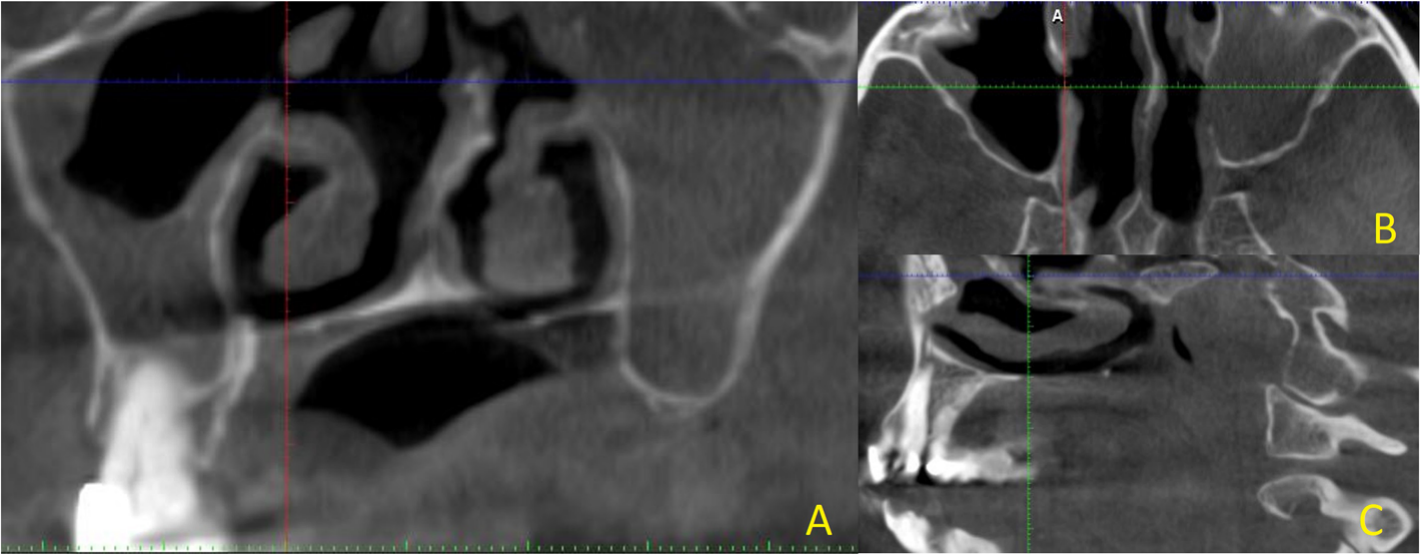
** A** coronal CBCT section showing AMO in the right maxillary sinus, deviation of the nasal septum and mucosal thickening in the floor of the sinus. **B** corresponding axial and **C** sagittal section. Coloured linear annotations are used to precisely locate the site of AMO in all three planes

In order to identify and confirm the existence of the AMO, the examiners used coronal Fig. 1A, axial Fig. 1B and sagittal views Fig. 1C. Horizontal and vertical annotation overlays directed the radiologist to correctly display the AMO in all three planes. Inter-observer reliability was evaluated using Cohen Kappa test.

**Fig. 2:**
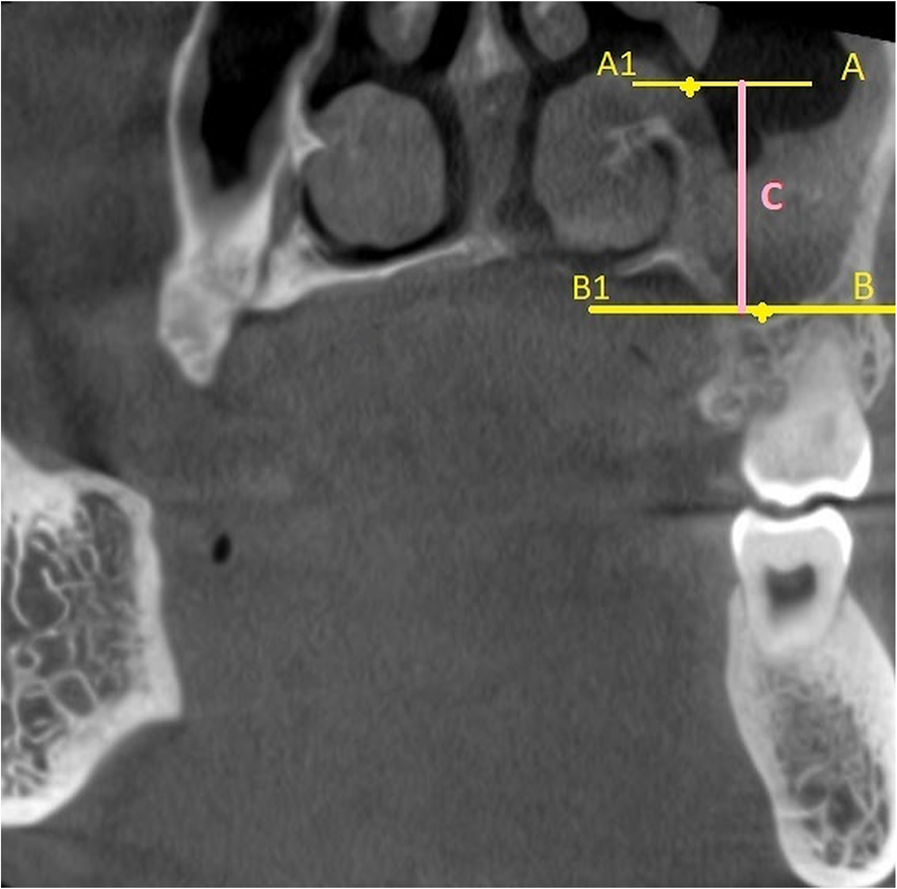
Coronal CBCT section showing method used to determine the location of AMO in vertical dimension (C). The distance C is obtained by measuring the distance between 2 horizontal lines A1-A (passing through the most inferior point of the AMO) and B1-B (passing through the most inferior point on the floor of the maxillary sinus)

**Fig. 3:**
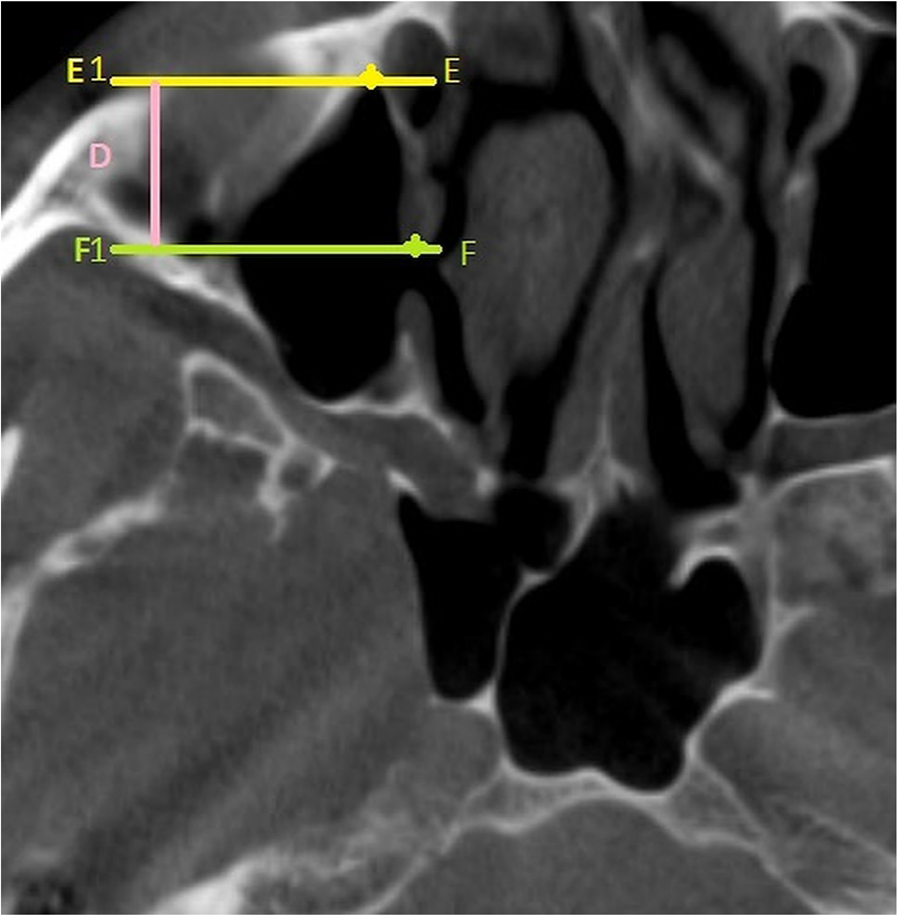
Axial CBCT section showing method used to determine the location of AMO in antero-posterior dimension (D). The distance (D) is obtained by measuring the distance between 2 horizontal lines F1-F (passing through the most anterior point of the AMO) and E1- E (passing through the most anterior point of the sinus)

**Fig. 4:**
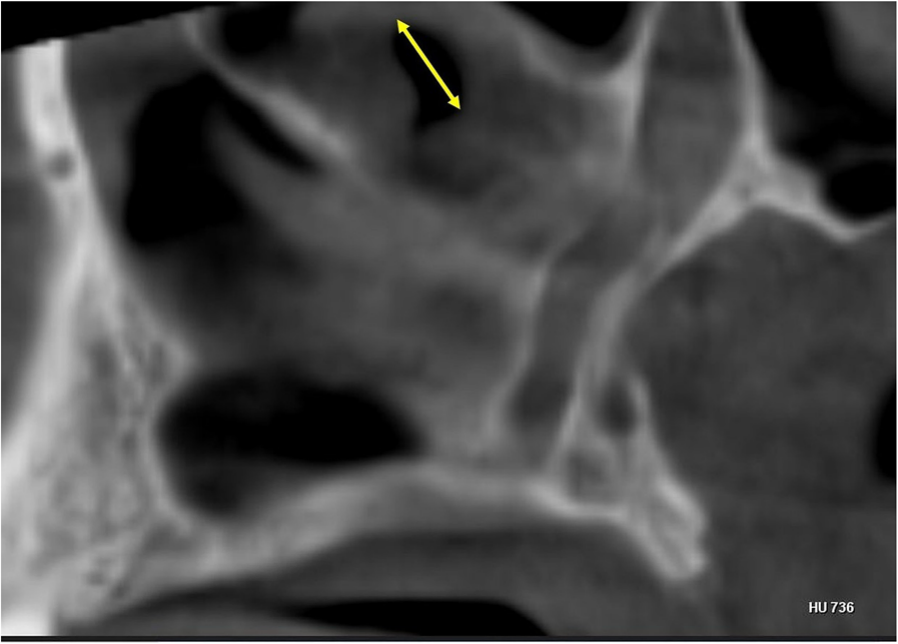
Two-sided yellow arrow depicting the maximal length of the AMO located on the lateral nasal wall observed in the sagittal CBCT section

The vertical and antero-posterior measurements for the location of AMO was determined by using the technique suggested by Butaric et al. [9]. The distance of the AMO from the floor of the sinus (C) was calculated using the coronal CBCT sections, by measuring the distance between 2 horizontal lines A1-A (passing through the most inferior point of the AMO) and B1-B (passing through the most inferior point on the floor of the maxillary sinus) Fig. 2. The antero-posterior location of the AMO was evaluated on the axial CBCT sections by measuring the distance (D) between 2 horizontal lines F1-F (passing through the most anterior point of the AMO) and E1- E (passing through the most anterior point of the sinus) Fig. 3. The longest dimension of AMO in the sagittal CBCT segment was measured as suggested by Hung et al. Fig. 4 [10].

**Fig. 5:**
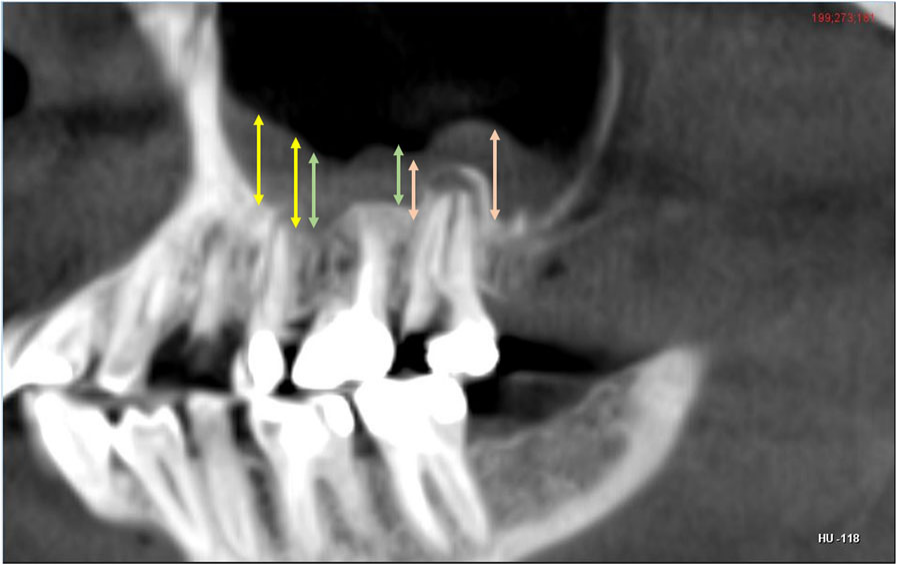
Sagittal section showing the points at which the mucosal thickening was measured in dentate study subjects. The mesial and distal sides of the second premolar (yellow arrows), first molar (green arrows) and second molar (pink arrows) were the six points of measurement

**Fig. 6:**
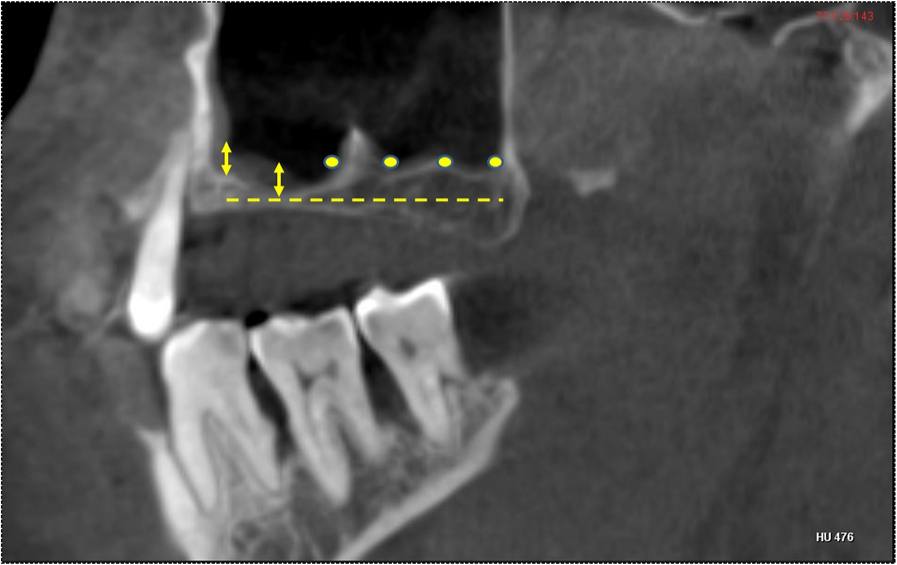
Sagittal CBCT section showing the points at which measurements were made in a edentulous study subject. Six equidistant points (arrows and dots) on a line (yellow discontinuous) connecting the anterior and posterior most points on the floor of the sinus in sagittal section. (Dots indicate areas with no MT)

The MT was evaluated and classified based on criteria used by Sheiki et al. [11]. According to the classification, MTs were classified as Type 1 (< 1 mm), Type 2 (1–3 mm), Type 3 (3-6mm), Type 4 (6-10mm), Type 5(> 10 mm). According to the criteria the MT is measured at six points in each of the sinuses. The mesial and distal sides of the second premolar and first and second molar teeth were the six points of measurement. Among these six points, the highest point of the thickened mucosa of the sinus floor was considered to be the representative value of MT for that sinus Fig. 5. In the edentulous cases measurement of MT was performed at six equidistant points on a line connecting most of the anterior and posterior points on the sinus floor in the sagittal section Fig. 6. Only MT in the floor of the sinus was considered in our study.

### Data analysis

The data obtained regarding the occurrence, anatomical location, maximal length of AMOs and their association with MTs were evaluated using IBM SPSS statistics (Version 22, Armonk. NY: IBM Corp). Chi Square test and Fisher Exact Test were used to determine difference among study groups., Spearman’s Rho was used, to evaluate the association between the study groups.

## Results

In the present study, 400 maxillary sinuses from a total of 200 CBCT scans were evaluated for the presence of AMO. Among the 200 CBCT scans 131 (262 maxillary sinuses) belonged to male subjects and 69 scans (138 maxillary sinuses) belonged to female subjects. The age range of the subjects in the present study was between 22 and 65 years with the mean of 41.32 years. On evaluation of the dental status, revealed that 157 subjects were dentate, 40 were partially dentate and 3 were completely edentulous.

Two oral radiologists evaluated the presence, location and characteristics of the AMOs using multiplanar views of CBCT scans. The inter observer reliability using Cohens Kappa test was 0.83. We used the rubrics by Regier et al. 2012 for kappa rating [11]. The intra- observer reliability for detecting the presence of AMO was 0.85 for the oral radiologist A and 0.79 for B.

AMOs were found in 142 of the 400 maxillary sinuses. Primary maxillary ostium [PMO] was patent in 392 (98 %) of the 400 maxillary sinuses. A total of 142 AMOs were found, 90 AMOs were found in male subjects and 52 in female subjects. There was no statistically significant gender difference in the occurrence of AMO [OR = 1.1306, *P* = 0.54 (95 % CI: 0.7596 to 1.6827).

There was a radiographic evidence of obstruction of the PMO in eight maxillary sinuses. Among the eight maxillary sinuses with PMO obstruction, six sinuses showed the presence of AMO whereas two did not reveal the presence of AMO. The maximal length of the AMOs in the sagittal section varied from 0.5mm to 7mm. The AMOs were categorised into Class I, II and III based on their maximal length. On evaluation of the AMOs, 73 (51.40 %) were less than 2 mm in length (Class I), 56 (39.43 %) were 2–4 mm in size (Class II), whereas 13(9.15 %) were greater than 4mm in size (Class III).

MT was observed among 132 of the 400 maxillary sinuses that were evaluated. When maxillary sinuses with AMOs (*n* = 142) were evaluated for the presence of MT, 96 maxillary sinuses (67 %) had evidence of MT, while 36 maxillary sinuses (33 %) had no radiographic evidence of MT. When the maxillary sinuses without AMOs (*n* = 258) were examined, it was found that 36 maxillary sinuses had radiographic evidence MT, while 222 sinuses had no radiographic evidence of MT. In sinuses with AMOs, there was a significantly higher occurrence of MTs [ *P* < 0.0001 OR = 0.2064 (95 % CI: 0.1337 to 0.3187).

**Fig. 7:**
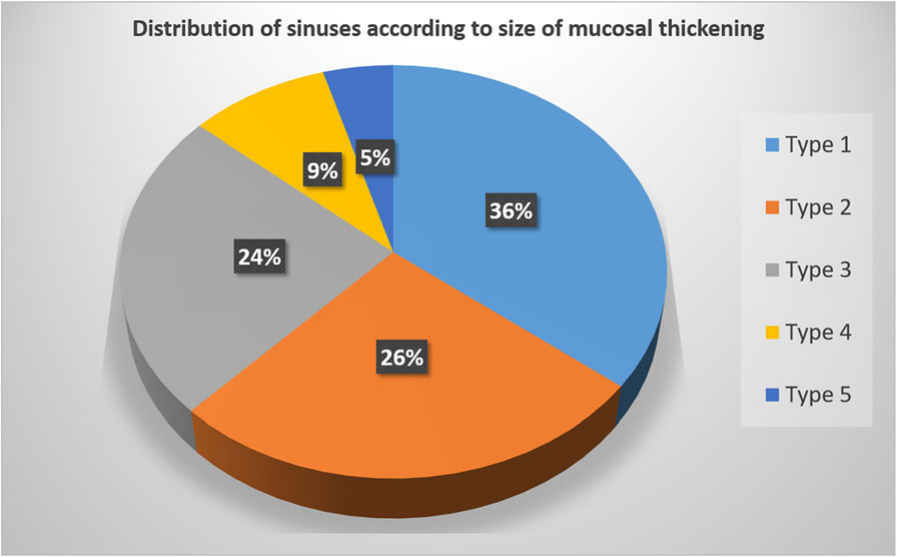
Distribution of the types of mucosal thickenings in maxillary sinuses of study subjects

**Fig. 8:**
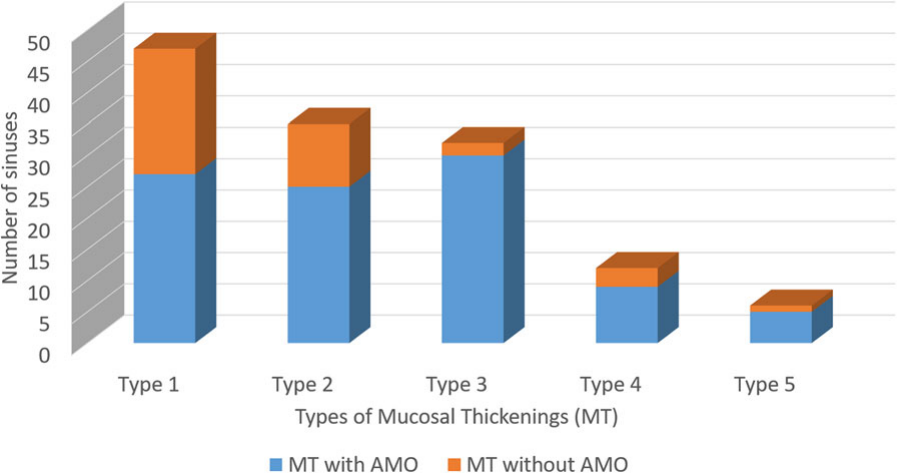
Occurrence of AMOs in different types of mucosal thickenings as per Sheiki et al. classification

When the size of MTs were categorized according to criteria defined by Sheiki M et al., it was noted that 47 (35.6 %) sinuses belonged to Type 1, 35 (26.5 %) sinuses belonged to Type 2, 32 (24.2 %) sinuses belonged to Type 3, 12 sinuses belonged to Type 4 and 6 sinuses belonged to Type 5. Figure 7. Type 3 MT had the highest occurrence of AMOs followed by types 1, 2, 4 and 5. Figure 8.

There was a significant (*P* = 0.006) association between the occurrence of AMOs and types of MT (Table 1). On post hoc comparison, **t**here was a significant difference in the occurrence of AMOs between Type 1 and Type 3(*p* < 0.001), Type 4(*p* < 0.001), Type 5(*p* < 0.001). Similarly, there was a significant difference in occurrence of AMOs between MT of type 2 and Type 3(*p* = 0.02), Type 4 (*p* = 0.05), Type 5 (*p* = 0.05). However, there was no statistically significant difference in the occurrence of AMOs between MT of Type 1 and 2 (*p* = 0.07). Similarly, there was no statistically significant difference in the occurrence of AMO between MT of Types 3 and Type 4 (*p* = 0.24), Type 4 and Type 5(*p* = 0.07) (Table 2). No significant correlation was observed between the occurrence of AMOs and type of MT was observed (r_s_ = -0.7, *P* = 0.18812).

**Table 1:** Overall comparison of the AMO among different types of MT as per Sheiki et al’s classification

Types of MT	AMO		Total sinuses	Fisher’s Exact Test
	**Present**	**Absent**		***p*-value**
**No MT**	36 (13.4 %)	232(86.5 %)	268 (100 %)	0.006*
**Type 1**	27 (57.4 %)	20 (42.6 %)	47 (100 %)	
**Type 2**	25 (71.4 %)	10 (28.6 %)	35 (100 %)	
**Type 3**	30 (93.8 %)	2 (6.3 %)	32 (100 %)	
**Type 4**	9 (75 %)	3 (25 %)	12 (100 %)	
**Type 5**	5 (83.3 %)	1(16.7 %)	6 (100 %)	

*p < 0.05 Statistically Significant, *p* > 0.05 Non-Significant, NS

**Table 2:** Post hoc comparison of the occurrence of AMOs among different types of MT as per Sheiki et al’s classification

Type of MT	Type 2	Type 3	Type 4	Type 5
**Type 1**	0.19(NS)^a^	< 0.001*^a^	< 0.001*^a^	< 0.001^b^
**Type 2**		0.02*^a^	0.05^b^	0.05^b^
**Type 3**			0.12(NS)^b^	0.41(NS)^b^
**Type 4**				1.00(NS)^b^

^a^Chi Square test

^b^Fisher Exact Test

*p < 0.05 Statistically Significant, *p* > 0.05 Non-Significant, NS

The mean distance from the most inferior point on the floor of the sinus to the inferior border of AMO in the coronal CBCT sections was 19.93 ± 1.68 mm. On overall and post hoc comparison, there was no significant difference in the mean distance among the different types of MT (Tables 3 and 4). There was no significant correlation between the mean distance of AMO from the sinus floor and type of MT (r_s_ = 0.3, *P* = 0.62384).

**Table 3:** Overall comparison of the mean distance between the floor of the sinus to the AMO in different types of MT as per Sheiki et al’s classification

Types Mucosal Thickening	Number of sinuses in each group	Mean distance of the AMO from the floor of the sinus in coronal CBCT section (mm = millimetres)	Fisher’s Exact Test
		**Mean(mm)**	***p*-value**
**No mucosal thickening**	36	18.95 ± 2.45	0.32
**Type 1**	27	20.23 ± 1.06	
**Type 2**	25	18.56 ± 2.13	
**Type 3**	30	21.85 ± 1.93	
**Type 4**	9	19.07 ± 0.78	
**Type 5**	5	20.96 ± 1.78	
**Total**	132	19.93 ± 1.68

**Table 4:** Post hoc comparison of the mean distance of the AMO from the sinus floor among different types of MT as per Sheiki et al’s classification

Type of MT	Type 1	Type 2	Type 3	Type 4	Type 5
**No MT**	0.07(NS)^a^	1.26(NS)^a^	0.07(NS)^a^	0.13(NS)^b^	0.3(NS)^b^
**Type 1**		0.26(NS)^a^	< 0.17(NS)^a^	< 0.62(NS)^b^	1.00(NS)^b^
**Type 2**			0.08(NS)^a^	0.24(NS)^b^	0.07(NS)^b^
**Type 3**				0.41(NS)^b^	1.00(NS)^b^
**Type 4**					0.07(NS)^b^

^a^Chi Square test

^b^Fisher Exact Test

*p* < 0.05 Statistically Significant, *p* > 0.05 Non-Significant, NS

The mean distance of the anterior most point of AMO to the anterior most point of the maxillary sinus in the axial section was 15.39 ± 1.82 mm. Overall comparison and post hoc comparison revealed that there was no significant difference in the mean distance of the AMO among different types of MT on (Tables 5 and 6). There was no significant correlation between the distance of AMO from the anterior-most point sinus and type of MT (r_s_ = -0.5, *P* = 0.391).

**Table 5:** Overall comparison of the mean distance between the anterior most point of the maxillary sinus to the AMO among different types of MT as per Sheiki et al’s classification

Types of Mucosal Thickening	Number of sinuses in each group	Mean distance of the AMO from the anterior most point in the maxillary sinus in axial section (mm = millimeters)	Fisher’s Exact Test
		**Mean (mm)**	***p*-value**
**No mucosal thickening**	36	14.32 ± 1.75	0.63
**Type 1**	27	16.54 ± 2.06	
**Type 2**	25	15.27 ± 1.05	
**Type 3**	30	14.92 ± 1.86	
**Type 4**	9	16.07 ± 2.01	
**Type 5**	5	15.22 ± 2.21	
**Total**	132	15.39 ± 1.82

**Table 6:** Post hoc comparison of the mean distance of the AMO from the anterior wall of the maxillary sinus among different types of MT as per Sheiki et al’s classification

Type of MT	Type 1	Type 2	Type 3	Type 4	Type 5
**No MT**	0.07(NS)^a^	1.26(NS)^a^	0.07(NS)^a^	0.13(NS)^b^	0.3(NS)^b^
**Type 1**		0.26(NS)^a^	< 0.17(NS)^a^	< 0.62(NS)^b^	1.00(NS)^b^
**Type 2**			0.08(NS)^a^	0.24(NS)^b^	0.07(NS)^b^
**Type 3**				0.41(NS)^b^	1.00(NS)^b^
**Type 4**					0.07(NS)^b^

^a^Chi Square test

^b^Fisher Exact Test

*p < 0.05 Statistically Significant, *p* > 0.05 Non-Significant, NS

A statistically significant (*P* = 0.001) association was observed between the maximal length of AMOs and the type of MT. Class 1 AMOs were predominantly found with Type 3 MT, Type 2 MT and no MT. Class 2 AMOs were predominant in Type 1 and Type 2 MTs. Class 3 were predominant in Type 4 and 5 MTs (Table 7).

**Table 7:** Association and between the classes of AMO as per Hung et al’s classification and types of MT as per Sheiki et al’s classification

	Length of AMO			Total
	**Class 1**	**Class 2**	**Class 3**	
**No Mucosal thickening (MT)**	24	19	3	46
	52.2 %	41.3 %	6.5 %	100.0 %
**Type 1- Mucosal thickening**	10	16	1	27
	37.0 %	59.3 %	3.7 %	100.0 %
**Type 2- Mucosal thickening**	14	10	1	25
	56.0 %	40.0 %	4.0 %	100.0 %
**Type 3- Mucosal thickening**	27	2	1	30
	90.0 %	6.7 %	3.3 %	100.0 %
**Type 4- Mucosal thickening**	2	2	5	9
	22.2 %	22.2 %	55.6 %	100.0 %
**Type 5- Mucosal thickening**	1	2	2	5
	20.0 %	40.0 %	40.0 %	100.0 %
**Fishers Exact test**	< 0.001*			

## Discussion

The main findings of this study revealed that the AMOs are likely to be located at approximately 19 mm from the inferior most point on the floor of the sinus. The maxillary sinuses with AMOs, showed significantly greater frequency of MTs. AMOs with greater maximal length were associated with higher degrees of MT. However, the location of the AMOs, were not affected by the degree of MT.

To discuss the clinical relevance and findings of our study it is important to note that maxillary sinus is an anatomic structure located in a vital location with close proximity to nasal cavity and the roots maxillary posterior teeth [12]. Maxillary sinuses are often associated with anatomic variations particularly the osteo-meatal complex which predisposes them to disease conditions [13]. AMO is one among the variations of the osteo-meatal complex. It is important to note that sinus disease does not necessarily mean the presence of osteo-meatal variation [14]. The term AMO was first coined in the year 1993 by Rice and Scheaffer, as a terminology for all the openings on the lateral nasal wall, other than a single primary ostium. [15]. It is not very clear whether AMO is congenital or acquired. Some researchers believe that AMOs usually occurs after an episode of acute maxillary sinusitis [16]. Some recent studies have highlighted the “recirculating mucus ring” phenomenon in which mucus circulation takes place between the normal ostium and the maxillary sinus AMO [3]. Apart from routine imaging procedures nasal endoscopy has been used for the detection of AMO [17, 18].

In our study, 35.5 % of the maxillary sinuses in the population were found to have AMOs. The prevalence of AMOs was examined in hospital / clinic settings using computerized tomography (CT) and endoscopy, while in anatomical research, cadavers were assessed. AMOs were present in 29.5 % of paranasal CT scans of the research cohort in one published study on the Jordanian population [7]. Recent CT based studies on AMOs reported a prevalence 19.1–46.3 % of the sinuses in Turkish population and 18 % in Indian population [6, 19, 20]. In another CT and endoscopy-based research on the Indian population conducted in 2018, AMOs were found in 23 % of the cohort [1]. Similar percentages were reported by purely endoscopic studies in the Indian population [18]. AMOs were reported in 13.8–26 % of the cadavers as per few recently study published anatomical studies. [15, 21–24]. Studies carried out on Chinese, Indian and Turkish population revealed prevalence rates of AMOs to be 47.2 %, 23.7 and 38.8 %, respectively [10, 25, 26]. In general, the prevalence of AMOs ranges from 20 to 50 % in studies using CT, CBCT scans and endoscopy. The likely explanations for this difference may be ethnic difference and the sensitivity of investigative imaging system. However, cadaveric studies have shown a lower prevalence of AMOs relative to studies using live subjects using CT, CBCT, or endoscopy. Post mortem anatomical distortion may be the likely cause for this lower prevalence [27]. Therefore, prevalence studies using imaging are more reliable.

Intra- and inter observer agreement is a vital issue in medical imaging interpretation and this must be assessed with the most suitable test for an accurate outcome of any imaging study [28]. In our study the inter observer reliability was 0.83 and intra-observer reliability for detecting the presence of AMO was 0.85. The observers’ reliability values in our study were consistent with the values found in the study by Hung et al. [10]. Nevertheless, few other researchers did not evaluate the components of inter and intra observer variability in their studies [6].

In our study there was no statistically significant difference in the occurrence of AMOs between male and female subjects, although the occurrence was numerically higher in male subjects. Similar observations were found in the studies by Bani-Ata et al. and Ghosh et al. [1, 7]. However, Hung K et al. [10] found AMOs to be more commonly present in, CBCT scans of female subjects. It is important to note that there was no statistical difference in terms of gender in any of the studies reiterating the fact that, gender had no significant influence on the occurrence of AMO [1, 7].

We found no statistically significant difference in the occurrence of AMOs when age was considered. The mean age of the research subjects was higher than that of the Hussein et al. and Sahin et al. studies [29, 30]. In most studies, the frequency of AMOs has not been significantly dependent on the age group [1, 6, 7, 17]. However, there was a higher prevalence of AMOs in older age groups in one study by Dedeoğlu N and Altun O. The authors attributed the greater incidence of AMOs in the elderly to be due to the age-related phenomenon and the resorption phenomenon that accompanied age-related edentulism [25].

In our study majority of the AMOs (90.83 %) were less than 4 mm in size. This was consistent with the findings of Hung K et al. [10]. Based on the maximal length, we divided the AMOs into three classes in our study. We also identified the location of the AMO based on its distance from the floor and the anterior wall of the maxillary sinus. The location of the AMO was determined by some of the studies and case reports based on the landmarks on the lateral nasal wall, such as the anterior and posterior fontanelle [1, 10, 31–33]. Other studies have identified the location of the ostium based on their distance from the landmarks such as floor of the sinus and anterior wall of the sinus [9, 34, 35]. Due to the variability of landmarks on the lateral nasal wall, we chose the latter approach based on measurements [36, 37]. In our study the height of the AMO from the floor of the sinus was 19.93 ± 1.68 mm. Radiographical studies have shown that the normal maxillary sinus height varies from 28 mm to 34 mm [38, 39]. Following the above measurements, AMOs are most likely to be found at a point between half and three-fourths of a line connecting the floor of the sinus to the roof. The fragility of the lateral nasal wall in this area might be the explanation for the incidence of AMOs at this site [40]. During inflammation, the maxillary sinus is almost half filled with inflammatory fluid in gel consistency. The cohesive forces prohibit normal ciliary transport of the fluid into the ostium, which is situated at a higher level. Therefore, the fluid content finds a point of structural fragility on the lateral nasal wall to escape the sinus [40].

MT is associated with collection of inflammatory fluid within the maxillary sinus [41–43]. Many recent studies have highlighted the relationship between periapical and periodontal health of maxillary dentition, sinus floor mucosa and maxillary sinusitis [44]. There are many approaches used by researchers to classify mucosal thickening in the maxillary sinus using CT and CBCT [45–47]. Magnetic resonance imaging (MRI) was also used in one of the earlier studies for the classification of MT in the maxillary sinus [48, 49]. MT has been graded by researchers either on the basis of thickening (mild/ moderate / severe, polyps, pseudocyst retention) or on the basis of numerical measurement ranges [11, 43]. We used Sheiki et al’s classification, which is based on measurements. In the present study, there was a statistically significant difference in the occurrence of AMOs when the thickening size exceeded 3 mm. It is important to note that, there is no consensus on the threshold values above which MT is considered to be pathological [50]. In our study, sinuses with radiographic evidence of MT, showed significantly higher occurrence of AMOs than those without such features. Similar results were obtained in a CT based study by Gusrov M et al., who suggested that AMO may be an accelerating factor in the transformation of sinus mucosal pathologies like retention cyst to antrochoanal polyps [19]. This association was also reflected in endoscopy-based and CBCT studies which revealed higher frequency of occurrence of AMOs in rhinosinusitis patients [17, 30, 51]. A CT-based analysis by Yenigun A et al. concluded that a probability of finding MT in the same sinus was correlated with the existence of AMOs. Additional observations from our study also suggest that the probability of finding an AMO during the radiographic evaluation of the maxillary sinus is higher if there is co-existing mucosal thickening of more than 3mm in the sinus floor. Such results suggest the Acquired Development Hypothesis model for the existence of AMO rather than the theory of congenital development. The length of the ostium plays a major role in the mucous circulation and thus will influence the mucosal thickening of the maxillary sinus. If the size of the AMO is up to 4 mm, the mucous secretions with a normal viscidness tend to circumvent the AMO in the maxillary sinus. In this situation the secretions do not pass through the AMO. However, the same phenomenon does differ for situations where the size of the AMO exceeds 4mm in diameter, whereby the mucous secretion portion of the mucous carpet flowing over the centre of the AMO flows into the centre of the meatus. The part of the mucous secretions in the margin of the AMO continues to pass along its borders of the AMO to finally reach the main natural ostium. The mucous secretions that have moved out of the maxillary sinus, through the main natural ostium, return to the same maxillary sinus when it makes a downward journey due to gravity and encounters AMO on its path. The secretions are laden with pathogenic micro-organisms from the nasal cavity layer during the re-entry process into the maxillary sinus. This malicious recirculation of secretions regularly aggravates the sinus condition, causing the sinus mucosa to pathologically thicken [2, 16].

Our study revealed that the MT did not have any statistically significant correlation with the location of the AMO. However, when we evaluated the maximal length the of AMOs with the type of MT, it was observed that larger AMOs were associated with higher degree of MT. This was similar to the observation in the study by Hung K et al. [10]. They proposed that AMO decreases the clearance of mucus secretions in the corresponding maxillary sinus, which could make the sinus susceptible to pathologies [6, 10].

Although the study establishes association between AMO and MT, there are some shortcomings that can be addressed in future studies. Future studies can be conducted with clinical and imaging findings, since clinical correlation is desirable to avoid over diagnosis of mucosal thickening based purely on imaging findings [42].

## Conclusion

The study findings indicate that the location of the AMO is independent of the degree of the MT. However, the occurrence of AMO is associated with degree of MT. Higher degrees of mucosa thickening are correlated with larger AMOs. The findings also show that there is a greater chance of discovering an AMO if the MT in the sinus is more than 3 mm. The findings of our study demonstrate the possibility of using CBCT to assess the occurrence and location of AMO in the osteo-meatal complex. It also encourages the use of CBCT in otolaryngology as a substitute for CT for imaging osteo-meatal complex with comparatively lower doses of radiation.

## Supplementary Information

**Additional file 1.**

## Data Availability

Submitted as supplementary material. Also available at figshare; doi 10.6084/m9.figshare.13385141
